# What is the evidence on the added value of implant‐supported overdentures? A review

**DOI:** 10.1111/cid.13027

**Published:** 2021-07-15

**Authors:** Thomas Van de Winkel, Laura Heijens, Stefan Listl, Gert Meijer

**Affiliations:** ^1^ Department of Oral Maxillofacial Surgery Radboud University Medical Center Nijmegen the Netherlands; ^2^ Department of Dentistry Radboud University Medical Center Nijmegen the Netherlands; ^3^ Department of Dentistry – Quality and Safety of Oral Healthcare Radboud University Medical Center, Radboud Institute for Health Sciences Nijmegen the Netherlands; ^4^ Section for Translational Health Economics, Department of Conservative Dentistry Heidelberg University Heidelberg Germany

**Keywords:** cost‐effectiveness, edentulous, implants, Oral Health Impact Profile, oral health‐related quality of life, overdenture

## Abstract

**Background:**

Implant‐supported overdentures (IODs) have been reported to increase patients' oral health‐related quality of life (OHRQoL) in comparison with conventional dentures (CDs); however, the conclusiveness of evidence on the clinical effectiveness and value for money of IODs versus CDs remains unclear.

**Purpose:**

To review how the added value of IODs is demonstrated in the literature.

**Materials and methods:**

MEDLINE, EMBASE, and the Cochrane Database were searched for randomized control trials, controlled clinical trials, and prospective cohort studies containing evaluations of the economic and health benefits and costs of IODs. Information about the clinical effectiveness, such as magnitude of bite forces or chewing efficacy, OHRQoL, costs, and cost‐effectiveness of IODs, was extracted.

**Results:**

A total of 17 articles were included, reporting 15 economic evaluations: 11 cost‐utility analyses (CUAs), 2 of which were combined with a cost‐effectiveness analysis (CEA), and 2 cost–benefit analyses (CBAs). Seven CUAs used the Oral Health Impact Profile (OHIP) questionnaire while four used satisfaction questionnaires to assess the OHRQoL. One study applied quality‐adjusted prosthesis years (QAPYs) for this purpose. The CBAs expressed both the beneficial outcome and the costs of the IOD in monetary terms. The included studies employed a large variety of economic evaluation methods, which limited cross‐study comparability.

**Conclusions:**

On the basis of existing economic evaluations, IODs have frequently been suggested to be a cost‐efficient treatment alternative to CDs; however, the comparability between the various economic evaluation studies was limited due to the different outcome measures used. In addition, it remains unclear whether the additional health benefits of IODs outweigh the higher costs. This is largely dependent on the decision maker's valuation of oral health outcomes. Future research is encouraged to further elucidate patient willingness to pay for IODs and the societal return on investing in IODs more generally.

**What is known**

- Implant‐supported overdentures (IODs) increase clinical effectiveness: bite forces and chewing efficacy increase.
- Implant‐supported overdentures (IODs) also increase patients' oral health‐related quality of life (OHRQoL) as compared to conventional dentures (CDs).

**What this study adds**

- The existing evidence on the added value of IODs and the methodologies used were reviewed.
- Cost‐effectiveness analyses (CEAs) and cost‐utility analyses (CUAs) are the proper instruments to calculate the incremental costs (IOD versus CD) in relation to the incremental health improvement.

## 1 INTRODUCTION

Edentulism (being toothless) can lead to significant functional impairment, as well as unfavorable esthetic and psychological changes in patients. Reported drawbacks include restrictions in diet and a limited ability to eat certain foods,^1^ speech impairment, and the loss of support for facial musculature, which has an aging effect on appearance.^2^ Edentulism is even classified as a physical handicap by the World Health Organization.^3^

Installing dental implants has the potential to mitigate these drawbacks. Many articles corroborate that implant‐supported overdentures (IODs) provide significantly higher satisfaction levels, quality of life, and better mastication than mandibular conventional dentures (CDs).^4^, ^5^, ^6^, ^7^, ^8^, ^9^, ^10^ As a result of these positive findings, since 2002 it has been recommended that, in case of lack of retention, a mandibular IOD retained by two interforaminal implants (IOD‐2) should be considered the first treatment choice.^11^ Because of the palate as substantial bearing surface, the CD remains the first step in prosthetic rehabilitation for the edentulous maxilla. Nevertheless, the success of IODs in terms of stability, function, speech, and patient satisfaction has also been shown for the upper jaw.^11^, ^12^, ^13^ An extra advantage of the presence of functioning implants is that clinically significant progressive bone loss is prevented.^14^ Disadvantages, however, are the invasive treatment, need for maintenance, high costs, and risks for peri‐implantitis.

Despite their benefits, IODs also incur higher treatment costs than CDs, leading to the question of whether IODs provide a reasonable value for money. An economic evaluation means “ensuring that the value of what is gained from an activity outweighs the value of what has to be sacrificed.”^15^ Such economic calculations can inform patients, healthcare providers, insurers, and policy makers about IOD value for money.^16^, ^17^, ^18^ In order to determine whether the benefits produced by a particular program exceed the opportunity costs of providing that program, a reliable method of measuring and comparing outcomes is required.^19^ After all, the diversity in included cost‐categories, the various types of economic evaluation used and the different interpretations of it, may complicate the drawing of firm conclusions.

Beneficial aspects for patients can be expressed in terms of clinical effectiveness, such as the number of prosthetic complications, the magnitude of bite forces in newtons, or measuring the masticatory efficacy. In contrast, patient‐reported outcome measures (PROMs) describe patient's perceived health benefits in qualitative terms; for example, patient satisfaction is often scored with the aid of questionnaires asking about general satisfaction and/or masticatory ability with different food types. Another way to identify PROMs is to measure the oral health‐related quality of life (OHRQoL). In dentistry for this purpose the Oral Health Impact Profile (OHIP)‐list is often used.^20^

Various types of economic evaluation have been presented.^21^, ^22^ Both cost‐effectiveness analyses (CEAs) and cost‐utility analyses (CUAs) calculate the incremental costs of a specific treatment in relation to the incremental health improvement. CEAs describe clinical effectiveness, such as number of prosthetic complications or magnitude of bite forces in newtons. CUAs are typically expressed in natural (qualitative) units such as OHRQoL, life years gained, or quality‐adjusted life years (QALYs). Both CEAs and CUAs rely on the incremental cost‐effectiveness ratio (ICER), which compares the difference in costs against the health improvement associated with two or more treatment alternatives.^17^, ^21^ CEAs and CUAs are especially suitable for interventions that are more effective than their alternatives but also cost more. In cost–benefit analyses (CBAs), both health outcomes and costs are expressed in monetary terms, thus enabling a direct comparison. For example, it has long been recommended to assess patient preferences in terms of “willingness to pay” (WTP) for different treatments, such as implant placement.^23^

Conceptually similar to WTP is the concept of WTA (“willingness to accept”), in which patients are asked which amount of money they would accept to go back to their baseline situation, for example, from their IOD to their CD. For nonpatients, this is the maximum amount that they are willing to receive to forgo implant therapy. WTP/WTA are thought to be important in health technology assessments by providing insight into the impact that the risks and benefits of treatments have on society.^24^

The purpose of this study was to review the existing evidence on the added value of IODs and the methodologies used. It was hypothesized that the information, as available in so far literature, is too diverse to draw firm conclusions.

## 2 MATERIALS AND METHODS

Thomas Van de Winkel and Laura Heijens conducted a search of the literature written in English and published between January 1995 and August 2020 that compared health outcomes to the involved costs with respect to an IOD. Special attention was focused on the relationship between costs and the extent of the increased OHRQoL.

The MEDLINE, EMBASE, and the Cochrane Database were screened using the following terms: (economic evaluation) and (dental implant) and overdenture. As the search results were minimal, it was decided to choose for the more general terms: cost and (dental implant) and overdenture. As methodology the PICO Principle was used (Table 1).

**TABLE 1:** The PICO (population, intervention, control, and outcomes) format as strategy for the research question

PICO principle	
Population	Edentulous patients
Intervention	Treatment with IOD
Comparison	CD (new or pre‐existing)
Outcomes	(1) Health benefits, such as satisfaction, chewing capacity, OHRQoL (2) Costs, (3) value for money

Inclusion criteria: for this review, only studies that focused on economic evaluations while providing information about both benefits in OHRQoL and costs of IODs were included, meaning CEAs, CUAs, and CBAs.

Exclusion criteria: case reports, articles which were written in a language other than English, or those involving patients who still had natural teeth were excluded.

Systematic reviews and meta‐analyses obtained from the database search were subsequently perused for other papers on this topic (snowballing). The selected articles were independently evaluated by two reviewers (Laura Heijens and Thomas Van de Winkel). In case of disagreements about inclusion, a consensus discussion was conducted. If no consensus could be reached, Gert Meijer took the final decision. A Cohen's kappa analysis was calculated to determine the interevaluation reliability of the articles included between the two evaluators.^25^

### 2.1 Quality assessment

For each selected article, the 24‐item checklist of the Consolidated Health Economic Evaluation Reporting Standards (CHEERS) was used to evaluate whether each item was met. As the aim of the CHEERS list is to optimize the reporting of health economic evaluations, only the quality of reporting is judged, not the quality of conduct.^21^, ^22^ The selected randomized controlled trials (RCTs) and clinical controlled trials (CCTs) were evaluated by two independent reviewers (Laura Heijens and Thomas Van de Winkel). In case of disagreement, first a discussion took place to come to a mutual agreement. If no agreement was reached, a final decision was made by Gert Meijer.

To further appraise the risk of bias and the methodological approach of the selected RCTs and CCTs, the respective studies were evaluated by two independent reviewers (Laura Heijens and Thomas Van de Winkel) using Cochrane Risk of Bias Tool.^26^ Again, in case of dispute, discussions were held to reach agreement, which, if unsuccessful, was followed by a final decision of Gert Meijer. The articles were screened for randomization, blinding of randomization, selective reporting, blinding of staff and participants, blinding of the results, and the presence of incomplete data. Studies were judged to have a “high risk of bias” if one of the items showed a high bias score. If one of the items had an “uncertain risk of bias,” but no “high risk of bias” on the other items, the study was considered as an “uncertain risk of bias.” In cases where all items scored a low risk of bias, the study was categorized as “low risk of bias.” Cohort studies were qualitatively assessed using Form III for assessing a cohort study by the Dutch Cochrane Center (2003). In addition, the articles were screened for the following confounding factors: whether the research was funded by the manufacturer (potential benefit), inclusion or exclusion criteria related to patient factors (disease, mental state), individual factors such as age and number of dental implants, and date of publication with reference to costs.

### 2.2 Data extraction

For the selected articles, it was first noted if a CEA, CUA, or CBA was included. Furthermore, the following items were recorded: authors, year of publication, inclusion and exclusion criteria regarding the health of the participants, number and age of the participants, follow‐up period, number and location of the implants, outcome measures, and the raw data and conclusions about the increase in OHRQoL. Specifically, information was gathered about the type of costs, such as for IOD fabrication and costs incurred by loss of working time due to travel and attending treatment sessions.

For analytical purposes, it was also noted in which year and in which currency the costs were presented. All raw data were converted to the same currency (US dollars; USD) using the exact exchange rate of the year in which the investigation was performed to allow an optimal comparison.^27^ If the year in which the costs were incurred was not clear, the relevant author of the article was consulted.

## 3 RESULTS

**FIGURE 1:**
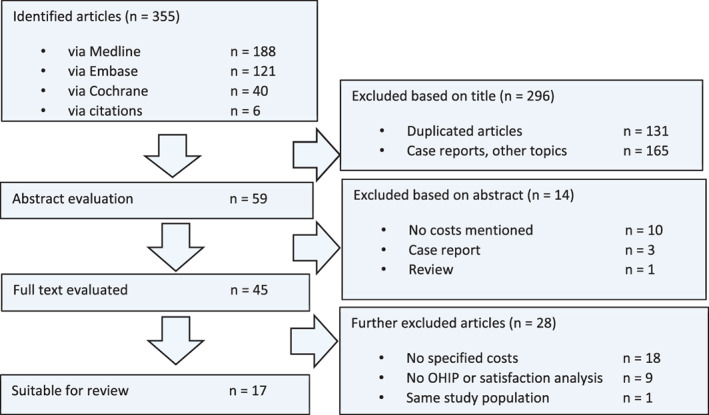
The search strategy used to identify the 17 articles to be reviewed

A total of 355 studies were identified based on the search terms. After the first screening, which consisted of reading titles and abstracts, 59 and 45 articles were selected, respectively. After intensively evaluating the relevant 45 articles, it appeared that some publications lacked important data, or described the same patient population. Ultimately, 17 studies remained that were suitable for analysis (Figure 1). With respect to the inclusion of the selected articles, between the two reviewers a substantial agreement (Cohen's kappa: 0.72) was measured.^25^

### 3.1 Characteristics of included studies

In Tables 2 and 3, the characteristics of all 17 included studies are presented, comprising five RCTs,^28^, ^29^, ^30^, ^31^, ^32^ five CCTs,^33^, ^34^, ^35^, ^36^, ^37^ two cohort studies,^38^, ^39^ and five economic evaluations.^40^, ^41^, ^42^, ^43^, ^44^ For a single study population in the Netherlands, Timmerman and colleagues reported the mandibular IOD satisfaction score^32^ and Stoker and colleagues the mandibular IOD costs.^31^ Taking these articles together, this randomized study can be labeled a CUA. The same accounts for the two studies presented by Wetzels and colleagues; functional benefits were described in 2016, and costs in 2017. As such, they provided a CEA/CUA analysis of the installation of a mandibular IOD‐2 in patients who have been treated for oral cancer.^35^, ^36^

**TABLE 2:** Characteristics of the included studies

Author	Study type	Type of analysis/Score of CHEERS list	Patients (*n*)	Follow‐up period (months)	Short description of study	Outcome reported
Alfadda and Attard^33^	CCT	CUA 18 (24) 75%	75	168	Cost analysis for IOD; immediate versus conventional loading	Total costs, OHIP‐20, ICER
Attard and colleagues^34^	CCT	CUA 14 (24) 58%	77	12	Clinical costs, PROMS for immediate‐loading protocol for IOD	Total costs, OHIP‐20, ICER
Della Vecchia and colleagues^28^	RCT	CUA 17 (24) 71%	120	6	Cost‐effectiveness analysis of IOD. MDI versus conventional implants	Total costs, OHIP‐EDENT, ICER
Heydecke and colleagues^29^	RCT	CUA 19 (24) 79%	60	60		Total costs, OHIP‐20, cost‐effectiveness
Jawad and colleagues^30^	RCT	CEA/CUA 14 (24) 58%	46	6	Cost‐effectiveness analysis of IOD‐2 or IOD‐4 MDIs versus conventional implants	Total costs, OHIP‐20, cost‐effectiveness. Chewing capacity
Listl and colleagues^40^	EE	CUA 20 (24) 83%	833	120	Cost‐effectiveness analysis of 6 and 4 implants in the edentulous maxilla	Manufacturing, maintenance cost, CEAC
Matthys and colleagues^38^	Cohort study	CUA 10 (24) 42%	56	60	Maintenance cost ratios of locators	Cost ratio OHIP‐14
Matthys and colleagues^39^	Cohort study	CUA 17 (24) 71%	116	60	Initial and maintenance costs for Locator versus Dalla Bona ball implants	Cost‐effectiveness plane, OHIP‐14
Stoker and colleagues^31^ and Timmerman and colleagues^32^	RCT RCT	CUA 15 (24) 63%	110	96	Cost analysis for three types of IOD in lower jaw	Total costs
			110	12	Satisfaction for three types of IOD in lower jaw	Satisfaction questionnaire
Wetzels and colleagues^35^ and Wetzels and colleagues^36^	CCT CCT	CEA/CUA 16 (24) 67%	193	60	Outcome of two implants installed during ablative surgery (DAS protocol) or postponed (P protocol)	Costs, chewing capacity, satisfaction questionnaire
Zitzmann and colleagues^37^	CCT	CUA 18 (24) 75%	60	36	Cost‐effectiveness analysis of two different IODs and CD	Total costs and ICER (QAPYs)

Abbreviations: CCT, controlled clinical trial; CEA, cost‐effectiveness analysis; CEAC, cost‐effectiveness acceptability curve; CUA, cost‐utility analysis; EE, economic evaluation; ICER, incremental cost‐effectiveness ratio; OHIP, Oral Health Impact Profile; PROM, patient‐related outcome measure; RCT, randomized clinical trial; QAPY, quality‐adjusted prosthesis years.

**TABLE 3:** Characteristics of the economic evaluation (EE) studies describing WTP

Author	Type of analysis/score of the 24‐items CHEERS list	Patients (*n*)	Follow‐up period (months)	Short description of study	Outcome reported
Esfandiari and colleagues^41^	CBA 12 (24) 50%	36	24	WTP/WTA IOD‐2	Satisfaction score (VAS 0–100) 61% (*n*): WTP: $3399 USD^a^ 89% (*n*): WTP: $3399 USD^b^ in case of monthly payments 92% (*n*): WTA: priceless
Sendi and colleagues^42^	CBA 15 (24) 63%	16	60	WTP/WTA IOD‐2	Satisfaction (VAS 1–10) WTP: 4971 USD^b^ WTA: 26157 USD^b^
Srivastava and colleagues^43^	CBA 12 (24) 50%	38	N/A	(partially) dentate were interviewed WTP/WTA IOD‐2	WTP: $5481 USD^c^ WTP: $171 USD^c^ as one‐time payment, with 20% chance of becoming edentulous WTA: priceless
Srivastava and colleagues^44^	CBA 12 (24) 50%	317	N/A	WTP/WTA (partially) dentate were interviewed about IOD‐2	WTP: $5348 USD^d^ WTP: $27 USD^d^, as monthly payment with a 20% chance of becoming edentulous WTA: priceless

Abbreviations: CBA, cost–benefit analyses; CHEERS, Consolidated Health Economic Evaluation Reporting Standards, WTA, willing to accept; WTP, willing to pay.

^a^ Esfandiari and colleagues^41^ used 2008 Canadian dollars (CAD) in their article (1 CAD = 0.9441 USD).

^b^ Sendi and colleagues^42^ used 2013 Swiss francs (CHF) in their article (1 CHF = 1.0793 USD).

^c^ Srivastava and colleagues^43^ used 2011 Canadian dollars (CAD) in their article (1 CAD = 1.0114 USD).

^d^ Srivastava and colleagues^44^ used 2012 Canadian dollars (CAD) in their article (1 CAD = 1.0002 USD).

In total, nine CUAs^28^, ^29^, ^31^, ^33^, ^34^, ^37^, ^38^, ^39^, ^40^ were presented, plus two combinations of CEA/CUA^31^, ^32^, ^35^, ^36^ and four CBAs.^41^, ^42^, ^43^, ^44^ The scores of the 24‐item CHEERS checklist varied between 10 (42%) and 20 (83%).^22^

Of the RCTs and CCTs, seven studies presented a “high risk of bias,”^28^, ^30^, ^33^, ^34^, ^35^, ^36^, ^37^ as did the two cohort studies.^38^, ^39^ An “uncertain risk of bias” was noted for three studies.^29^, ^31^, ^32^ In summary, the quality of all included RCTs, CCTs, and cohort studies was debatable or low.

### 3.2 CEA/CUA: Study design

In total, four studies presented a follow‐up period varying between 6 month and 1 year and involved real spend costs.^28^, ^29^, ^30^, ^34^ Calculated costs were also reported over a 5‐year period^39^ and over 8 years.^31^, ^32^ The only long‐term study involving real costs followed patients for a 14‐year period.^33^ Although Heydecke and colleagues presented costs over an even longer period (17.9 years), real costs were not included, but calculated based on the Delphi group opinion technique, using an annual price increase of 3%–5%.^29^, ^45^ The same accounts for the study of Zitzmann and colleagues: they collected financial data over a 3 years period and estimated costs for a 10‐year period also using an annual price increase of 3%–5%.^37^ List and colleagues calculated costs that were based on the German private dental insurance fee.^40^ Within this system, providers' fees can be adjusted by different factors corresponding to the treatment complexity (factor 1: low complexity; factor 2.3: average complexity; factor 3.5: high complexity). One study reported maintenance costs as a percentage of the initial costs.^38^

Most studies compared the IOD to the original CD.^28^, ^30^, ^31^, ^32^, ^35^, ^36^, ^37^, ^38^, ^39^, ^40^ In two studies, patients were divided into groups in which patients received a new CD or a IOD‐2.^29^, ^37^ In two other studies, first a new CD was manufactured. Subsequently, a few months later implants were installed and the IOD‐2 delivered.^33^, ^34^

Most studies focused on IOD's on conventional implants, two studies concentrated on IODs on MDI's.^28^, ^30^ All but one study addressed IODs in the lower jaw. Solely, Listl and colleagues calculated costs for an IOD‐4 versus IOD‐6 in the upper jaw.

With respect to the aims, two studies compared an IOD‐2 to a CD,^29^, ^37^ two studies compared conventional versus immediate loading,^33^, ^34^ and one group compared two surgical protocols in oncology patients.^35^, ^36^ And the others compared two treatment modalities, for example, MDIs versus conventional implants,^28^, ^30^ ball attachment versus locators,^39^ and ball attachments versus bar attachment.^31^, ^32^

### 3.3 CEA/CUA: Total costs

In Table 4, the “total costs” of various CD and IOD types for the edentulous lower jaw are shown.

**TABLE 4:** Type of construction in relation to total costs

Type of construction	Time period	Total costs: initial + maintenance + complication + recall + travel time
CD in mandible	1 year	$1385^a^; Heydecke and colleagues^29^
	17.9 years	$3801^a^; Heydecke and colleagues^29^
	3 years	$2242^b^; Zitzmann and colleagues^37^
IOD on two mandibular implants	14 years	$4349^c^; Alfadda and Attard^33^ (conventional loading)
		$4022^c^; Alfadda and Attard^33^ (immediate loading)
	1 year	$1983^d^; Attard and colleagues^34^ (immediate loading)
		$1779^d^; Attard and colleagues^34^ (conventional loading)
	0.5 year	$566^e^; Della Vechia and colleagues^28^
	1 year	$2458^a^; Heydecke and colleagues^29^
	17.9 years	$5960^a^; Heydecke and colleagues^29^
	0.5 year	$1048^f^; Jawad and colleagues^30^
	5 years	$4716^g^; Matthys^39^ (ball attachment)
		$4302^g^; Matthys^39^ (locator attachment)
	8 years	$3683^h^; Stoker and colleagues^31^ (Dalla Bona ball)
		$3849^h^; Stoker and colleagues^31^ (bar construction)
	5 years	$3288^i^; Wetzels and colleagues^35^ (DAS protocol)
		$6108^i^; Wetzels and colleagues^35^ (P protocol)
	3 years	$5413^b^; Zitzmann and colleagues^37^
IOD on four mandibular implants	8 years	$4912^h^; Stoker and colleagues^31^ (bar construction)
	3 years	$10881^b^; Zitzmann and colleagues^37^
IOD on mandibular MDIs	0.5 year	$318^e^; Della Vechia and colleagues^28^ (two MDIs)
		$511^e^; Della Vechia and colleagues^28^ (four MDIs)
		$620^f^; Jawad and colleagues^30^ (two MDIs)
IOD‐4 in maxilla	10 years	$7494^j^; Listl and colleagues^40^ (IOD‐4)
IOD‐6 in maxilla		$8697^j^; Listl and colleagues^40^ (IOD‐6)

Abbreviations: CD, conventional denture; IOD, implant‐supported overdenture; MDI, mini dental implants.

*Note*: Conversion table^27^: https://www.ofx.com/en‐au/forex‐news/historical‐exchange‐rates/yearly‐average‐rates/.

^a^ Heydecke and colleagues used 1999–2000 Canadian dollars (CAD) in their article (1 CAD = 0.6733 USD).

^b^ Zitzmann and colleagues used 2000 Swiss francs (CHF) in their article (1 CHF = 0.61 USD).

^c^ Alfadda and Attard used 2016 Canadian dollars (CAD) in their article (1 CAD = 0.7551 USD).

^d^ Attard and colleagues used 2002 Canadian dollars (CAD) in their article (1 CAD = 0.6367 USD).

^e^ Della Vecchia and colleagues used 2014 Brazilian reals (BRL) in their article (1 BRL = 0.5720 USD).

^f^ Jawad and colleagues used 2017 British pound sterling (GBP) in their article (1 GBP = 1.288 USD).

^g^ Matthys and colleagues used 2020 Euros in their article (1 EUR = 1.1290 USD).

^h^ Stoker and colleagues used 2000 Euros (EUR) in their article (1 EUR = 1.0850 USD).

^i^ Wetzels and colleagues used 2008 Euros (EUR) in their article (1 EUR = 1.4713 USD).

^j^ Listl and colleagues used 2014 Euros (EUR) in their article (1 EUR = 13 292 USD).

Initial costs were low in Canada; $627 for a CD versus $1796 for an IOD‐2.^29^ In Switzerland initial prices were higher: $1540 for a CD and $4230 for an IOD‐2.^37^ It became clear that an IOD‐2 is 2–3 times more expensive than a CD in terms of initial costs.

After 1 year, Heydecke and colleagues^12^ calculated $1385 of total costs for a CD, which increased to $3801 after 17.9 years.^29^ For the IOD‐2 costs were $2458 after 1 year, which went up to $5960 in 17.9 years. Initially, an IOD‐2 was almost 3 times more expensive than a CD; however, after 17.9 years this ratio decreased to less than 2 times.^29^ Apparently, an IOD‐2 becomes relatively cheaper in time and, however, continues to be more expensive than a CD.^29^ This outcome was corroborated by Zitzmann and colleagues, who calculated that total costs after 3 years were $2242 and $5413, for a CD and IOD‐2, respectively, resulting in a ratio of 2.4.^37^

Although the phrase “total costs” was often used, the definitions of this term varied. Initial costs were calculated mostly on an individual basis including, if present, the national dental tariff structure for the purchase of the implants, costs of surgical treatment such as the salary of the clinical workers and supporting personnel, the use of the operating room, and medicines. Costs of the prosthodontic treatment were also included in this category, in addition to laboratory fees. “Maintenance costs” comprised the ongoing costs of the prosthodontic treatment and laboratory fees, such as remakes, relines, hardware replacement, and professional services provided by the prosthodontist and/or the surgeon. Sometimes, the costs of annual recall visits (“recall costs”) were included in the “maintenance costs.” Only few studies included “patient time costs,” corresponding to the loss of income from missing work due to treatment or traveling.^33^, ^34^, ^37^

### 3.4 CEA/CUA: Patient‐reported outcome measures

PROMs can be expressed using satisfaction questionnaires. For example, the McGill denture satisfaction questionnaire^46^ was applied by Della Vecchia and colleagues.^28^ Using a VAS scale (0–100 mm), the following variables were assessed: general satisfaction, ability to speak, and esthetics. In addition, the ability to chew five different foods was recorded: standard‐sized pieces (3 × 1 × 1 cm) of raw apple, bread, raw carrot, cheese, and dry sausage. An alternative is the Denture Satisfaction Scale (DSS),^47^ as executed by Attard and colleagues,^34^ which comprises 12 questions and is scored using a 5‐point Likert scale with the following categories: (1) *totally satisfied*, (2) *very satisfied*, (3) *reasonably satisfied*, (4) *not very satisfied*, and (5) *not at all satisfied*. Other authors compiled their own questionnaire with different numbers of questions and scales.^32^, ^35^, ^36^, ^37^, ^40^, ^41^, ^42^

To measure OHRQoL in dentistry, one of the Oral Health Impact Profile (OHIP)‐lists can be used, which focuses solely on toothless patients, such as OHIP‐14, OHIP‐EDENT, and OHIP‐20, which comprise 14, 19, and 20 questions, respectively.^48^ Similar to the original OHIP‐49, the OHIP‐20 and OHIP‐14 cover the same seven domains: functional limitation, pain, psychological discomfort, physical disability, psychological disability, social disability, and handicap.^20^ The responses are based on a Likert scale ranging from 0 for “*never*” to 4 for “*very often*,” meaning the maximum score for OHIP‐20 is 80; the lower the score, the higher the OHRQoL that is achieved.

The effect of treatment using the OHIP system as PROM is depicted in Table 5. The OHIP‐20 questionnaire was applied in four studies,^29^, ^30^, ^33^, ^34^ the OHIP‐14 in two studies,^38^, ^39^ and the OHIP‐EDENT in one study.^28^ Sometimes different Likert scales were used; for example, with total scores in the range of 0–80^29^, ^33^ or of 20–100.^34^ Others introduced their own OHIP‐20 version,^30^ a 6‐point Likert scale varying between 1 and 6, covering nine items: (1) *ease of cleaning*, (2) *general satisfaction*, (3) *ability to speak*, (4) *comfort*, (5) *esthetics*, (6) *stability*, (7) *ability to chew*, (8) *function*, and (9) *oral condition*.^30^

**TABLE 5:** Change in OHIP points as a result of a CD, IOD‐2, IOD‐4, IOD on two MDIs, IOD on four MDIs

Article	Mandibular IODs versus CDs: OHRQoL scored in three types of OHIP questionnaires; OHIP‐14, OHIP‐20, and OHIP‐EDENT			Effect in QAPYs
Alfadda and Attard^33^	CD “old” (baseline)	71 OHIP‐20 (Likert 0–4)		
	CD new	51 OHIP‐20 (Likert 0–4)		
	IOD‐2 “immediate” loading	28 OHIP‐20 (Likert 0–4; after 1 year) 25 OHIP‐20 (Likert 0–4; after 5 years) 34 OHIP‐20 (Likert 0–4; after 14 years)		
Attard and colleagues^34^	CD “old” (baseline)	71 OHIP‐20 (Likert 1–5)		
	CD new	50 OHIP‐20 (Likert 1–5)		
	IOD‐2	24 OHIP‐20 (Likert 1–5; after 1 year)		
Della Vecchia and colleagues^28^	CD “old” (baseline	14–18 OHIP‐EDENT (Likert 0–2)		
	IOD‐2	6 OHIP‐EDENT (Likert 0–2; after 0.5 years)		
	IOD‐2 on MDIs	3 OHIP‐EDENT (Likert 0–2; after 0.5 years)		
	IOD‐2 on MDIs	2 OHIP‐EDENT (Likert 0–2; after 0.5 years)		
Heydecke and colleagues^29^	CD “old” (baseline)	56 OHIP‐20 (Likert 0–4)		
	CD new	47 OHIP‐20 (Likert 0–4; after 1 and 17.9 years)		
	IOD‐2	31 OHIP‐20 (Likert 0–4; after 1 and 17.9 years)		
Jawad and colleagues^30^	IOD‐2	41 OHIP‐20 (Likert 1–6; after 0.5 years)		
	IOD‐2 on MDIs	56 OHIP‐20 (Likert 1–6; after 0.5 years)		
Matthys and colleagues^38^	CD “old”	20 OHIP‐14 (Likert 0–4; during intake)		
	IOD‐2	3 OHIP‐14 (Likert 0–4; after 1 and 5 years)		
Matthys and colleagues^39^	IOD‐2 (locators)	9 OHIP‐14 point reduction; after 5 years		
	IOD‐2 (ball attachment)	3 OHIP‐14 points reduction; after 5 years		
Zitzmann and colleagues^37^	IOD‐4	Dental health state preference VAS 0–1	CD “old” (baseline): 0.37	IOD‐4: 1.57
	IOD‐2		CD “old” (baseline): 0.35	IOD‐2: 1.46
	CD new		CD “old” (baseline): 0.52	CD new: 0.68

Abbreviations: CD, conventional denture; IOD, implant‐supported overdenture; MDI, mini dental implants; OHIP, Oral Health Impact Profile; QAPYs, quality‐adjusted prosthesis years.

The OHIP‐14 comprises 14 questions, each with a score between 0 (very positive) and 4 (very negative), resulting in a maximum score of 56. This was only used by Matthys and colleagues.^38^, ^39^

Della Vecchia and colleagues had a preference for the OHIP‐EDENT, which consists of 19 questions with answers on a Likert scale of 0–2, leading to a maximum score of 38.^28^ Only four domains were covered: masticatory discomfort, psychological discomfort, social disability, and oral pain/discomfort.^49^

As alternative measure for PROMs, Zitzmann and colleagues used QAPYs, which corresponds to functioning for 1 year in the best possible prosthetic state.^37^

### 3.5 CEA/CUA: Incremental cost‐effectiveness ratios

The cost‐effectiveness of an IOD on four to six maxillary implants was calculated in only one study.^40^ All others addressed an IOD in the lower jaw.^28^, ^29^, ^30^, ^31^, ^32^, ^33^, ^34^, ^35^, ^36^, ^37^, ^38^, ^39^

In a CEA or CUA always an ICER is presented. For a mandibular IOD‐2 versus a new CD, the ICER was $81 per OHIP‐20 point after 1 year^34^ and $152 per OHIP‐20 point after 17.9 years.^29^ Costs went up as the years passed: $129, $159, and $362 per OHIP‐20 point after 1, 5, and 14 years, respectively.^33^

Using the OHIP‐EDENT questionnaire, two studies proved that a mandibular IOD‐2 on two MDIs resulted in a lower ICER score than an IOD on conventional implants: $28 versus $47^28^ or $17 versus $39.^30^ Even an IOD‐4 on MDIs was cheaper than an IOD‐2 on conventional implants (ICER $38 vs. $47).^28^

Zitzmann and colleagues also calculated an ICER, but formulated the measured effect in QAPYs.^37^ The costs per QAPY were $5551 for an IOD‐2 versus $12078 for an IOD‐4 after 3 years. These amounts reduced in a 10‐year period to $2318 and $4331 for an IOD‐2 versus an IOD‐4, respectively (Table 6).

**TABLE 6:** Incremental cost‐effectiveness ratio (ICER) of a CD, IOD‐2, IOD‐4, IOD on two MDIs, IOD on four MDIs in the lower jaw in USD

Type of prosthesis in the lower jaw		Cost‐effectiveness presented as ICER
IOD‐2 implants	Versus new CD	$86^b^ per OHIP‐20 point after 1 year; Attard and colleagues^34^
		$152^a^ per OHIP‐20 point after 17.9 years; Heydecke and colleagues^29^
		$129^c^ per OHIP‐20 point after 1 year; Alfadda and Attard^33^ $159^c^ per OHIP‐20 point after 5 years; Alfadda and Attard^33^ $362^c^ per OHIP‐20 point after 14 years; Alfadda and Attard^33^
IOD‐2	Versus CD “old” (baseline)	$47^d^ per OHIP‐EDENT point after 0.5 year; Della Vecchia and colleagues^28^
IOD‐2 on MDIs		$28^d^ per OHIP‐EDENT point after 0.5 year; Della Vecchia and colleagues^28^
IOD‐4 on MDIs		$38^d^ per OHIP‐EDENT point after 0.5 year; Della Vecchia and colleagues^28^
IOD‐2	Versus CD “old” (baseline)	$39^e^ per OHIP‐EDENT point after 0.5 year; Jawad and colleagues^30^ $17^e^ per OHIP‐EDENT point after 0.5 year; Jawad and colleagues^30^
IOD‐2 MDIs		

Type of prosthesis in the lower jaw		Cost‐effectiveness presented QAPYs
IOD‐2 implants	Versus new CD	$5551^f^ per QAPY after 3 years; Zitzmann and colleagues^37^ $2318^f^ per QAPY after 10 years; Zitzmann and colleagues^37^
IOD‐4 implants		$12 078^f^ per QAPY after 3 years; Zitzmann and colleagues^37^ $4331^f^ per QAPY after 10 years; Zitzmann and colleagues^37^

Abbreviations: CD, conventional denture; IOD, implant‐supported overdenture; MDI, mini dental implants; OHIP, Oral Health Impact Profile; QAPYs, quality‐adjusted prosthesis years.

^a^ Heydecke and colleagues used 1999–2000 Canadian dollars (CAD) in their article (1 CAD = 0.6733 USD).

^b^ Attard and colleagues used 2002 Canadian dollars (CAD) in their article (1 CAD = 0.6367 USD).

^c^ Alfadda and Attard used 2016 Canadian dollars (CAD) in their article (1 CAD = 0.7551 USD).

^d^ Della Vecchia and colleagues used 2014 Brazilian reals (BRL) in their article (1 BRL = 0.5720 USD).

^e^ Jawad and colleagues used 2017 British pound sterling (GBP) in their article (1 GBP = 1.288 USD).

^f^ Zitzmann and colleagues used 2000 Swiss francs (CHF) in their article (1CHF = 0.61 USD).

### 3.6 CBA: Willingness to pay

All four included CBA studies (Table 3) focused on the costs of a mandibular IOD‐2. Three studies originated from Canada,^41^, ^43^, ^44^ while one was conducted in Switzerland.^42^

Esfandiari and colleagues^41^ interviewed patients who participated 2 years earlier in a RCT^8^ in which they all received a new CD in their upper jaw, combined with a new CD or an IOD‐2 in their mandible. The authors claimed that about 50% of the participants would pay 3 times more for a mandibular IOD‐2 ($3399) than for a CD ($1133). If payment in monthly installments was allowed, even 96% of the respondents stated that they would pay $3399 for an IOD‐2 (Table 3). An average of 5 years after the mandibular IOD‐2 installation, Sendi and colleagues conducted a telephone interview with the request to answer eight questions. Retrospectively, satisfaction rate was queried for the time of IOD‐2 delivery, after 6 and 24 months, and at the moment of the interview.^42^ The average WTP price for an IOD‐2 was $4971.

Both studies conducted by Srivastava and colleagues addressed patients who were still dentate. In the first study questionnaires were used, in the second study WTP data were collected through telephone interviews or internet‐based questionnaires. Both studies delivered the same WTP price (about $5500) in one payment on condition of a 90% success rate.^43^, ^44^ Patients are willing to prepay $171 as one‐time assurance premium for private dental insurance, meaning that they will be fully covered for a mandibular IOD‐2 if needed in the future based on a 20% chance of becoming toothless.^43^ In case of a 20% chance to become edentulous, the WTP was $27 as monthly payments for private insurance. The WTP was higher when household income or dental needs were higher.^44^

In short, both dentate participants,^43^, ^44^ as well as edentulous patients who have already been treated with implants^41^, ^42^ were asked about WTP. Patient WTP for an IOD‐2 on interforaminal implants varied from $3399^41^ to $4971.^42^

The WTA numbers are particularly interesting; when asked for how much money they would turn in their IOD‐2 and go back to their original CDs, five patients valued the IOD‐2 state from $26.157^42^ to priceless^41^, ^42^, ^43^, ^44^ (Table 3).

## 4 DISCUSSION

The findings of the present review indicated considerable variation in the type, reporting, and quality of economic evaluation studies on IODs in comparison with their baseline situation, which was always the existing or “old CD.” Different questionnaires, diverse definitions of costs versus health outcome calculation methods, and varying timeframes were applied. With respect to IODs, variations in availability and affordability, pricing policies, level of reimbursement, and the discount rate made a comparison of the selected studies difficult.

Checklists such as CHEERS are commonly used in reviews to standardize the assessment of quality or completeness with respect to the economic evaluations. There is some discussion about how to interpret such checklists; with respect to CHEERS, the minimum reported cutoff for an evaluation to be considered “high quality” was 63%, while the maximum cutoff was 94%.^22^

In our analysis, most CEA/CUA studies scored 63% or more, and thereby are judged at least as “acceptable.”^32^, ^33^, ^35^, ^36^, ^37^, ^39^, ^40^, ^41^, ^43^, ^44^, ^46^

Three studies scored between 42% and 58%; their contribution was “average” or “low.”^30^, ^34^, ^38^

Except for one study (Sendi and colleagues^46^), scores for CBAs were 50% illustrating that their contribution was “average.”^41^, ^43^, ^44^

### 4.1 CEA/CUA: Total costs

In Table 4, the “total costs” of various CD and IOD types for the edentulous lower jaw are shown.

Initial costs were low in Canada; $627 for a CD versus $1796 for an IOD‐2.^29^ In Switzerland initial prices were higher: $1540 for a CD and $4230 for an IOD‐2.^37^ It became clear that an IOD‐2 is 2–3 times more expensive than a CD in terms of initial costs.

After 1 year, Heydecke and colleagues^12^ calculated $1385 of total costs for a CD, which increased to $3801 after 17.9 years.^29^ For the IOD‐2 costs were $2458 after 1 year, which went up to $5960 in 17.9 years. Initially, an IOD‐2 was almost 3 times more expensive than a CD; however, after 17.9 years this ratio decreased to less than 2 times.^29^ Apparently, an IOD‐2 becomes relatively cheaper in time and, however, continues to be more expensive than a CD.^29^ This outcome was corroborated by Zitzmann and colleagues, who calculated that total costs after 3 years were $2242 and $5413, for a CD and IOD‐2, respectively, resulting in a ratio of 2.4.^37^

### 4.2 Initial costs/total costs

Costs of interventions are not limited to the initial treatment, but also include costs for follow‐up care, maintenance, complications, and patient time lost due to the performance of the treatment working and traveling. In addition, total costs need to be assessed over time. As such, costs should be discounted by an annual set rate.^18^ Taking all these factors in account, an IOD‐2 is initially 2–3 times more expensive than a CD in terms of initial costs. After 17.9 years this ratio decreased to less than 2 times.^29^, ^37^

Costs may differ significantly between patients and between healthcare systems; therefore, economic evaluations should be interpreted in the context of such cost structures. To allow comparison between studies, costs must at least be specified thoroughly within the environment in which they have been conducted.

### 4.3 Patient‐reported outcome measures

In health‐related economic evaluations of treatments, QALYs are used to express the gain both in the quality and length of life. For nonfatal conditions however, alternatives can be applied, such as satisfaction scores, OHIP indices, or QAPYs.^50^, ^51^ However, these differing evaluation methods cannot be compared to QALYs.^52^

With respect to satisfaction questionnaires, current score lists vary in scoring methodologies: some perform a VAS score, usually on a scale of 0–10 or 0–100, while others use a Likert scale to display their results. The use of the same questionnaire and the same scoring method enables the direct comparison of different studies. It is also crucial to realize that VAS scores are not ratio scale measurements. This implies that, for example, a difference between satisfaction scores of 20 and 40 is not comparable to a difference between scores of 70 and 90.^53^

A popular method is the use of OHIP question lists; however, these lists are not mutually comparable, and the scale also differs. To interpret, for example, an exact OHIP‐20 score, it is essential to know if a scale of 0–80 or 20–100 was used; however, differences calculated in OHIP‐20 points remain scale‐independent.

As the OHIP‐14 ranges from 0 to 56, and the OHIP‐EDENT from 0 to 38, the results described for the various outcomes are incomparable. To still allow comparison, the concept of a “minimum important difference” (MID) was introduced, which indicates the number of OHIP points that reflect a significant improvement. Locker and colleagues determined the MID for OHIP‐14, which was 5 scale points, or approximately 10% of the scale range of 56 points.^54^ For the OHIP‐20, a MID‐range was defined between 7 and 10, with a guide value of 9.^55^

When comparing the “old” CD (71 OHIP‐20 points) versus a mandibular IOD‐2 after 1 year (28 OHIP‐20 points), an improvement of 43 points was detected, which is more than 4 times the MID of 9 points.^33^ The same trend was seen in the study by Attard and colleagues; an improvement of 47 points was detected for an IOD‐2 versus the “old” CD.^34^ In comparison with a new CD, 16‐point^29^ or 26‐point^34^ differences in OHIP‐20 points were detected for the mandibular IOD‐2, which is about 2–3 times the MID value of 9 points.

Della Vecchia and colleagues reported in OHIP‐EDENT points.^28^ Although the MID was not established for the OHIP‐DENT, it could be set to four points using the 10% rule. In comparison with the “old” CD, an improvement of 16 points for the IOD‐2 on implants, of 11 points for the IOD‐2 on MDIs, and of 13 points for the IOD‐4 on MDIs was reported, all of which were about 3–4 times the MID; thus, similar to the level of change detected when using one of the other OHIP lists.^28^ In OHIP‐14 points, Matthys and colleagues presented an improvement of 16 points, which was more than 3 times the MID value of 5 points.^38^

In short, reported increases in OHRQoL are similar, regardless if the OHIP‐14, OHIP‐20, or OHIP‐EDENT methodology was used. Also, in QAPYs, a mandibular IOD‐2 yielded a satisfaction score 4 times higher than the “old” CD (0.35 vs. 1.46).^37^

### 4.4 Economic evaluations (ICERs)

With respect to CUAs delivering ICERs, four studies delivered an ICER comparing IODs with a new CD.^29^, ^33^, ^34^, ^37^ Another four studies compared IODs with an existing CD.^28^, ^30^, ^38^, ^39^

Heydecke and colleagues compared “between” two groups receiving either a new CD or a mandibular IOD‐2.^29^ The ICER for the IOD‐2 versus the new CD after 17.9 years was $152.^29^ Attard and colleagues measured “within” groups; first new dentures were made, then immediate‐loaded implants were installed and attached to the CDs to deliver an IOD‐2.^34^ After the first year they produced a lower ICER ($86), as no maintenance and recall costs were involved.

As the years go by, absolute costs increase over time due to continuous maintenance costs: $129 per OHIP‐20 point after 1 year, $159 per OHIP‐20 point after 5 years, and $362 per OHIP‐20 point after 14 years.^33^ To illustrate the extra value of an ICER, compared with a new CD, a maximum of $362 per OHIP‐20 point for the mandibular IOD‐2 was paid over a 14‐year period.^33^ In light of the reported improvement of 17 OHIP points over the full 14 years, this represents a total amount of $6154 after 14 years (or $440 per year).

Over the long term, Heydecke and colleagues reported lower costs.^29^ After 17.9 years they presented an ICER of $152 per OHIP‐20 point versus the new CD. With respect to the assessed decrease of 16 OHIP points, this translates to $136 per year for 17.9 years.^29^

The higher ICER ($362) and higher annual costs ($440) can be explained by the fact that Alfadda and Attard included the actual maintenance costs, while Heydecke and colleagues made an assumption about the long‐term costs using the Delphi group opinion technique.^29^, ^33^

In the short observation period of 0.5 years, it was concluded that a mandibular IOD‐2 on MDIs ($28 per OHIP‐EDENT point) was more cost‐effective than an IOD‐2 on conventional implants ($47 per OHIP‐EDENT point).^28^ This conclusion was corroborated by Jawad and colleagues, who presented a value of $17 per OHIP‐EDENT point for an IOD‐2 on MDIs versus $39 per OHIP‐EDENT point for an IOD‐2 on conventional implants.^30^ In both studies, solely edentulous individuals who wore clinically acceptable maxillary and mandibular CDs were included, and only the price of the implants and the attachment system, not the prosthetic costs, were included.

Using the method of calculating QAPYs, “total costs” were calculated for each QAPY. For a mandibular IOD‐2, $5551 per QAPY after 3 years was calculated and $2318 per QAPY after 10 years.^37^ The latter is in line with the $2432 calculated by Heydecke and colleagues.^29^ Also applying the QAPY methodology, costs are observed to decrease significantly over time.

WTP values for a mandibular IOD‐2 varied between $3399 and $5481^41^, ^44^ while WTA values varied from $26157^42^ to priceless,^41^, ^43^, ^44^ thereby underlining the beneficial effect of an IOD. As WTP surveys patient preferences for an IOD in monetary terms, not only is the appreciation of the IOD itself reflected, but in a way it also clarifies how a patient may endure discomfort, inconvenience, and a loss of time.^56^ Considerable bias can be introduced by misleading, ambiguous, or inappropriate questions, however.^57^

## 5 CONCLUSIONS

Total costs for a mandibular IOD‐2 were associated with 2–3 times higher total costs compared to a CD. Regardless of whether QAPYs or one of the OHIP lists was used, this resulted in a significant improvement in OHRQoL of about 2 times the MID in comparison with a new CD.

Although ICERs give an improved insight into the relationship between incremental costs and increases in OHRQoL, the comparability of the different economic evaluation studies is still complicated by the use of different outcome measures.

Using the same strategy to register outcomes and the same method to present costs would be helpful. However, uncertainty remains as to whether the additional health benefits of an IOD outweigh the higher costs, and this largely depends on the decision maker's valuation of oral health outcomes. As hypothesized, the information available in so far literature seems too diverse to draw firm conclusions. Future research is encouraged to enhance the comparability of oral health outcomes with overall health and wellbeing.

## CONFLICT OF INTEREST

The authors declare that there is no conflict of interest that could be perceived as prejudicing the impartiality of the research reported.

## AUTHOR CONTRIBUTIONS

Thomas Van de Winkel conceptualized the project idea, conducted the literature search, collected and analyzed the data, and drafted the manuscript. Laura Heijens and Gert Meijer contributed to the data analysis. Laura Heijens, Stefan Listl, and Gert Meijer critically reviewed and revised the manuscript.

## Data Availability

Data sharing is not applicable to this article as no new data were created or analyzed in this study.
